# Four months of DHA and whey protein supplementation in semi-professional female soccer players: a prospective non-randomized controlled study of omega-3 status, hematological outcomes, and physical performance

**DOI:** 10.3389/fnut.2026.1886305

**Published:** 2026-09-14

**Authors:** Svenja Nolte, Simon Klügel, Celina Maier, Sebastian Hacker, Karsten Krüger

**Affiliations:** Department of Exercise Physiology and Sports Therapy, Institute of Sports Science, Justus Liebig University Giessen, Giessen, Germany

**Keywords:** erythrocytes, female athletes, lipophilic index, omega-3, performance

## Abstract

**Introduction:**

Omega-3 polyunsaturated fatty acids (n-3 PUFAs) are well established in cardiovascular and cognitive health, yet their role in athletic performance remains insufficiently characterized. Mechanistic links have been proposed via erythrocyte membrane remodeling, blood rheology, and inflammatory modulation. Female athletes represent a particularly relevant but underexplored population, given the potential dual benefits for performance and menstrual health. This study aimed to determine whether long-term DHA plus whey protein supplementation improves erythrocyte omega-3 status, modulates hematological markers relevant to oxygen transport, and translates to performance or menstrual symptom benefits in female athletes. Menstrual symptoms and hormonal profiles were examined as exploratory secondary outcomes.

**Methods:**

In this prospective non-randomized controlled trial, 38 semi-professional female soccer players were assigned to either an encapsulated algae-based docosahexaenoic acid (DHA) intervention (1000 mg/day + whey protein; *n* = 20) or reference group (*n* = 18) over four months. Assessments were conducted pre-, mid- (2 months), and post-intervention, including blood analyses HS-Omega-3 Index (HS-O3I), lipophilic index (LI), hematology, cytokines and football-specific performance tests with menstrual cycle phase monitored and testing scheduled to account for hormonal fluctuations.

**Results:**

Baseline omega-3 status was suboptimal (HS-O3I: 5.08 ± 1.26%). DHA supplementation significantly increased the HS-O3I at Mid (6.36 ± 2.21%, *p* < 0.001) and Post (6.8 ± 2.7%, *p* < 0.001), with a large Group × Time interaction (ηp^2^ = 0.72). LI showed transient remodeling, with increases at Mid and partial normalization by Post. Hematological analyses revealed higher mean corpuscular volume (MCV) and reticulocyte hemoglobin in the intervention group, although baseline differences cannot be excluded. MPO concentrations declined over time (*p* < 0.05). No significant group differences occurred in sprint, jump, or individual anaerobic threshold (IAS) however, higher Post HS-O3I was associated with higher IAS (unadjusted R^2^ = 0.36, *p* < 0.05). Hormonal profile and premenstrual symptoms did not differ between groups.

**Discussion:**

Four months of DHA plus whey protein supplementation improved erythrocyte omega-3 status and may induce structural membrane changes. The association between higher HS-O3I and aerobic capacity might suggest a functional link between omega-3 status and endurance efficiency in female athletes, which still requires further confirmation.

## 1 Introduction

Omega-3 polyunsaturated fatty acids (n-3 PUFAs) have attracted increasing attention in sport science, with claims regarding their potential to enhance athletic performance becoming increasingly prominent. While their role in cardiovascular and cognitive health is well established, their translation into performance contexts is a relatively recent development, expanding from elite into amateur sports. This transition is largely driven by proposed benefits on recovery, adaptation, and oxygen transport (1, 2).

One hypothesized pathway is the modulation of erythrocyte deformability. Early studies demonstrated that red blood cell (RBC) flexibility is essential for efficient microvascular oxygen delivery and that exercise can impair this property (3, 4). Since n-3 PUFAs are incorporated into erythrocyte membranes, they may influence membrane fluidity, with potential consequences for blood rheology, oxygen transport, and ultimately performance capacity (5). However, despite this biologically plausible link, mechanistic insights into how membrane remodeling through omega-3 supplementation translates into functional outcomes remain limited.

Beyond hematological effects, n-3 PUFAs are discussed as modulators of inflammatory processes. Their incorporation into membrane phospholipids can alter the balance between arachidonic acid-derived pro-inflammatory eicosanoids and less potent EPA- and DHA-derived metabolites, thereby attenuating exercise-induced inflammation (6). Such effects may support recovery in athletes exposed to repeated high training loads (1). A previous meta-analyses suggest that a reduction of C-reactive protein (CRP), tumor necrosis factor-α (TNF-α), and interleukin-6 (IL-6) effects are most consistently observed at doses 1–3 g/day of combined EPA and DHA (7). Yet, the dose-response relationships and the distinct contributions of EPA vs. DHA remain poorly defined.

In nutritional strategies targeting athletes, adequate protein intake represents a critical co-factor for adaptation and recovery. High-quality proteins such as whey provide essential amino acids that facilitate muscle repair and remodeling. DHA may enhance the muscle's anabolic response to amino acids, suggesting potential synergy when combined with protein (8). This has been supported by findings in athletic populations, such as the study by Philpott and colleagues, where combined omega-3 and whey protein supplementation resulted in greater improvements in recovery and strength outcomes compared to omega-3 alone (9). Given this rationale, our intervention paired DHA with a whey protein base to support both membrane remodeling and training adaptation.

In female athletes, additional relevance emerges. Omega-3 supplementation has been linked to alleviation of premenstrual symptoms (10), suggesting a dual benefit in supporting both performance and female-specific health. Menstrual symptoms and hormonal profiles were therefore included as exploratory secondary outcomes, while cycle phase was standardized during testing to reduce hormone-related variability.

Despite this potential, most available research has focused on clinical or at-risk populations rather than athletic cohorts. For example, fatty fish consumption has been shown to reduce the lipophilic index (LI), an indicator of membrane rigidity (11), but the functional significance of LI for athletic populations remains underexplored. This gap is further reflected in reference values: the proposed optimal HS-O3I range of 8–11% has been derived from clinical outcomes (12), yet athletes consistently fall well below this threshold. Cross-study evidence indicates mean values of 4.4% in collegiate football players (13) and between 4–5% specifically in female athletes, as reported across independent cohorts of professional rugby (14) and mixed-sport elite athletes (15). Whether achieving this range confers tangible performance benefits in athletes remains an open question.

Taken together, there is a growing rationale for investigating the role of omega-3 PUFAs in sports performance, yet key gaps persist: (i) Can DHA supplementation in a whey protein formulation induce measurable remodeling of erythrocyte membranes in athletes, reflected in indices such as the HS-O3I and LI? (ii) Do such changes translate into alterations in hematological parameters relevant for oxygen transport? (iii) Are these adaptations associated with performance capacity in female athletes, and do exploratory analyses indicate relationships with menstrual-health outcomes?

In this study, we addressed these questions in a cohort of semi-professional female soccer players. We hypothesized that (1) 4 months of DHA supplementation in a whey protein formulation would significantly increase the HS-O3I and modify the lipophilic index, reflecting structural remodeling of erythrocyte membranes; (2) The omega-3-induced membrane remodeling will be associated with corresponding changes in hematological parameters relevant to oxygen transport; and (3) Omega-3 status following supplementation will be associated with performance capacity.

Associations between HS-O3I, PMS status, and hormonal profiles were examined exploratorily.

## 2 Methods

### 2.1 Participants and study design

A total of 38 semi-professional female football players from two regional clubs were enrolled in the PUFA (Performance Up-Leveling in Female Athletes) study, a prospective, non-randomized controlled intervention trial.

Eligible participants were female soccer players aged 18–35 years, competing at a semi-professional level and participating in regular team training (≥ 3 sessions per week). Only athletes free from acute or chronic diseases that could affect metabolism, performance, or hormonal status were included.

Exclusion criteria included known allergies or intolerances to study products (DHA, whey protein, or excipients), habitual high intake of fatty fish (> 2 portions per week), pregnancy or breastfeeding, medical conditions or medication use affecting lipid metabolism or hormonal function.

Participants were assigned to either the intervention group (*n* = 20) or the reference group (*n* = 18). Randomization was not applied to accommodate individual dietary preferences (e.g., vegetarian or vegan diets) and to promote adherence, as athletes with a greater willingness to consume the study product were allocated to the intervention group. The intervention period lasted 4 months. Athletes in the intervention group received a daily supplementation of 1,000 mg encapsulated algae-based docosahexaenoic acid (DHA FlexP 20) and 12 g Nutri^®^ Whey Isolate protein (powder formulation, delivered by FrieslandCampina, the Netherlands). The DHA-only formulation reflected the composition of the study product provided for the intervention; the study was not designed to compare DHA-only supplementation with a combined EPA+DHA protocol. All participants of the intervention group included in the analysis reported consuming the study product on at least 80% of the scheduled intervention days. The reference group did not receive any placebo supplementation, in line with the preference-based allocation approach and to avoid a supplementation burden in this subgroup. Accordingly, the study compared combined DHA-plus-whey supplementation with no supplementation and was not designed to distinguish DHA-specific effects from effects of whey protein or the combined supplementation strategy. Both groups were instructed to maintain their habitual diet, to abstain from additional omega-3 supplementation or major dietary and lifestyle changes throughout the study. To monitor potential changes in dietary omega-3 fatty acid intake over time, participants completed an omega-3 fatty acid food frequency questionnaire (n-3 FA FFQ) at the pre and post measurement point. This questionnaire was originally developed and validated as a screening tool to identify individuals with low omega-3 fatty acid intake in a Swiss population (16) and represents an adaptation of the validated U.S. Diet History Questionnaire from the National Cancer Institute (17). Following the intervention, participants were categorized according to data completeness at each assessment timepoint. Given the modest sample size, no formal intention-to-treat analysis with imputation of missing data was performed; all analyses were conducted on an available-case basis, using data from participants with valid measurements at the respective timepoint.

The study was registered with the Federal Institute for Drugs and Medical Devices (BfArM; DRKS00034542) and approved by the Ethics Committee of the University of Giessen (Approval No. 2024-0014). Written informed consent was obtained from all participants prior to enrollment, in accordance with the Declaration of Helsinki. Assessments were conducted at baseline (Pre, *n* = 38), after 2 months (Mid, *n* = 32), and after 4 months (Post, n = 27) (Figure 1).

**Figure 1:**
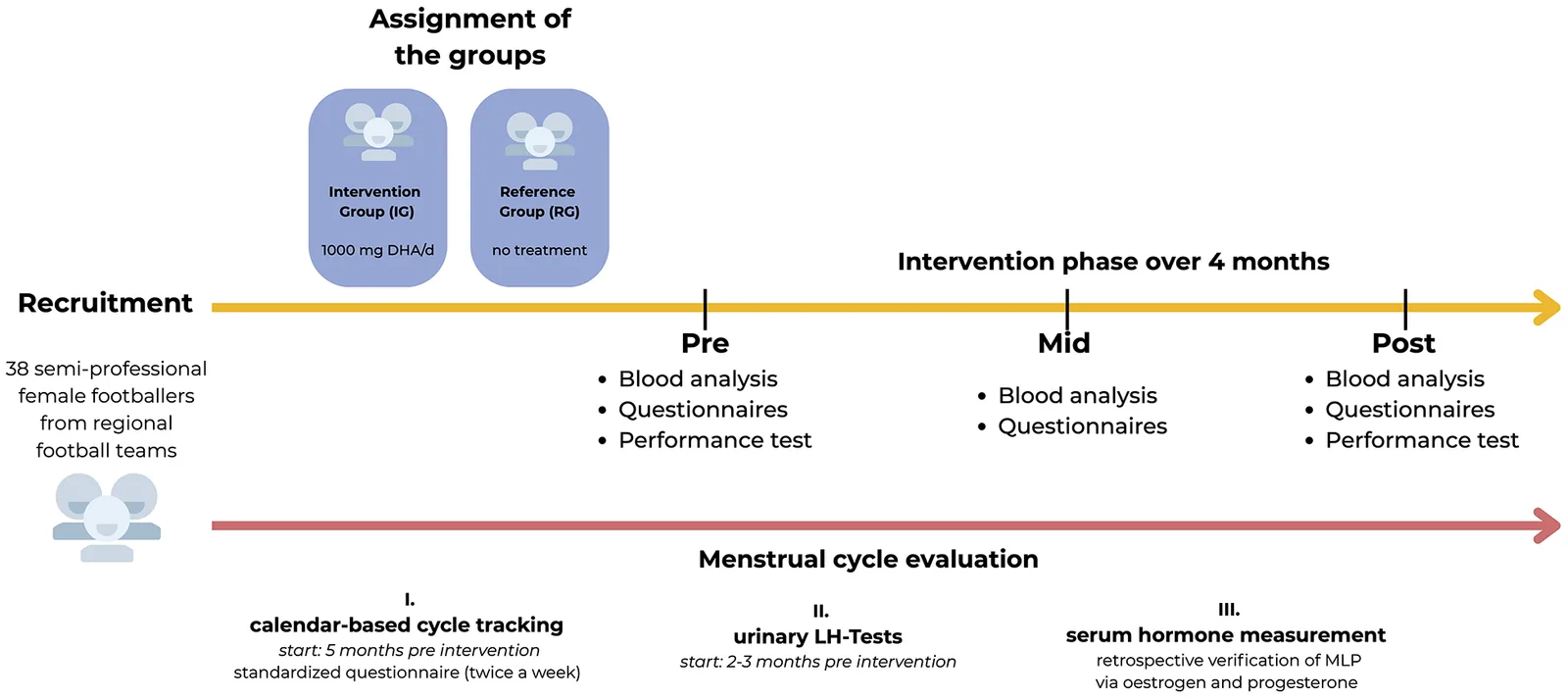
Study design of the PUFA study with three measurement time points, the 4 months intervention phase and the concurrently conducted cycle tracking.

*A priori* power analysis was performed using G^*^Power (version 3.1.9) to determine the required sample size. Assuming a large effect size (Cohen's d = 1.0), based on changes in the HS-O3I reported by Drobnic et al. (18) and Flock et al. (19) 16 participants per group (32 participants in total) were required to achieve a statistical power of 0.95 at a two-tailed α level of 0.05. To account for potential attrition and missing data, the recruitment target was increased by 25%, resulting in a planned sample size of 20 participants per group (*N* = 40).

Participant recruitment, allocation, follow-up, and analysis are summarized in Figure 2.

**Figure 2:**
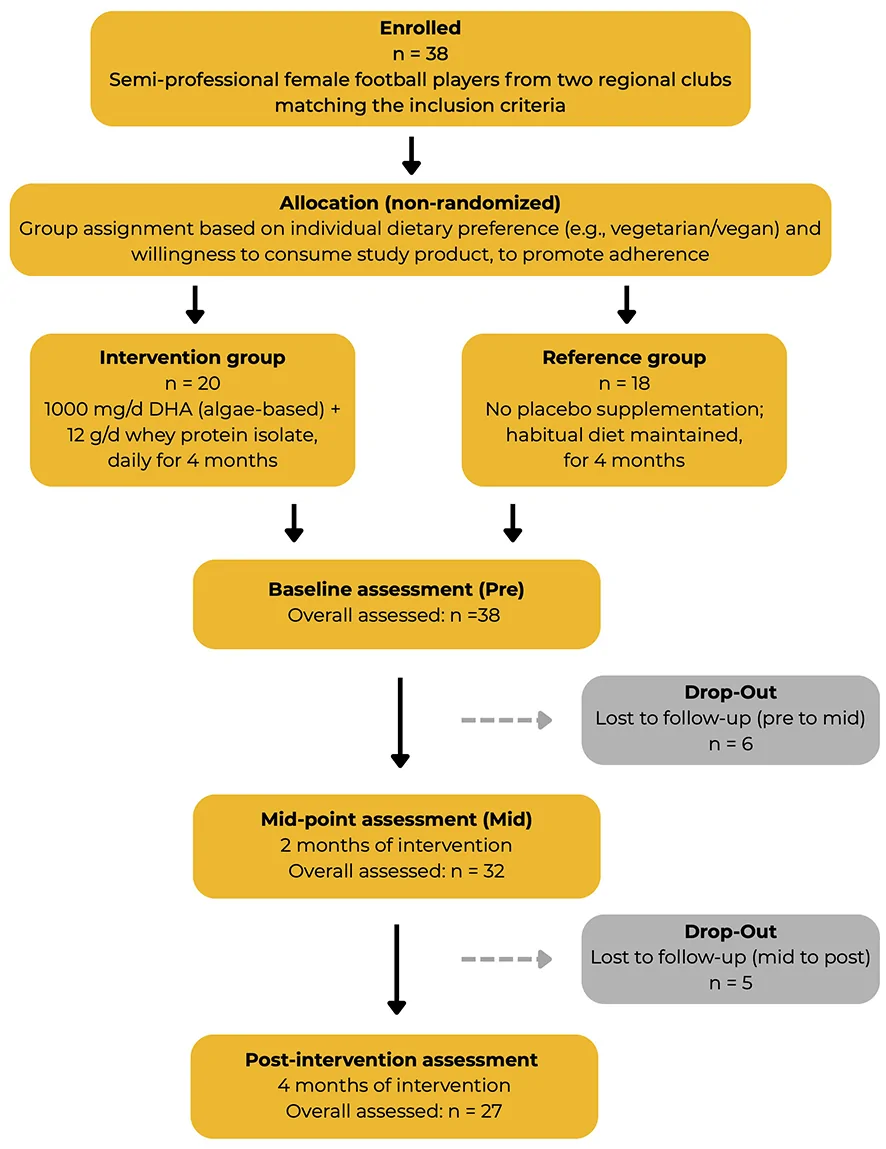
Participant flow through the PUFA study from enrolment to post-intervention assessment. DHA, docosahexaenoic acid; PUFA, Performance Up-Leveling in Female Athletes.

### 2.2 Menstrual cycle evaluation

To control for potential hormonal fluctuations, menstrual cycle tracking followed a strict three-step protocol as outlined by Peinado et al. (20). Initially, athletes completed a detailed questionnaire regarding their use of hormonal contraception or implants, age at menarche, and menstrual cycle regularity.

Beginning 5 months before the intervention, participants were trained in the symptothermal method for menstrual cycle tracking. They completed a standardized daily self-report form, recording cycle parameters such as menstruation onset and duration, basal body temperature, physical symptoms, cervical mucus consistency, and cervical position. This information was manually reviewed and summarized by the study team.

To ensure ecological validity and reflect real-world training conditions, all athletes were included irrespective of menstrual status or contraceptive use. This included athletes with a natural menstrual cycle, those who were not eumenorrheic by strict definition proposed by Elliott-Sale (21) and athletes using oral contraceptives or hormonal implants. For participants using combined oral contraceptives, measurements were scheduled during the active pill phase. This period is characterized by stable circulating levels of synthetic estrogen and progestin, which more closely resemble the hormonal milieu of the mid-luteal phase than the withdrawal (placebo) phase. This approach was chosen to minimize variability associated with hormonal fluctuations and to align measurement timing between naturally cycling and oral contraceptive users (21).

Following 3 months of cycle tracking, participants initiated urinary luteinizing hormone (LH) testing, guided by individualized notifications from the study team. LH test results were validated through submitted photographs, with positivity determined by the research staff. This combined approach (symptothermal method and LH testing) was maintained throughout the intervention phase to ensure accurate timing of physiological measurements within the menstrual cycle.

Pre, Mid and Post measurements (blood samples, exercise testing and questionnaires) for all participants were performed in the mid-luteal phase (MLP) or the active pill phase to ensure hormonal comparability.

### 2.3 Menstrual symptoms

Premenstrual symptom severity was assessed using the Premenstrual Symptoms Screening Tool (PSST), a validated self-report instrument designed to identify premenstrual disorders and evaluate the functional impact of cycle-related symptoms (22).

### 2.4 Blood sample collection

Venous blood samples were collected at all three measurement time points: Pre, Mid and Post. Blood draws were scheduled between 07:00 and 09:00 a.m. following an overnight fast. Participants were instructed to abstain from intense physical activity the day prior to sampling and to include at least one rest day after their most recent competition. On each sampling day, a brief questionnaire was administered to assess recent training load and current health status, to rule out acute illness or infection.

Samples were collected into the following tubes (Sarstedt, Nümbrecht, Germany): three 7.5 mL Serum Gel tubes, two 7.5 mL EDTA tubes, and one 2.7 mL EDTA tube. The 7.5 mL EDTA samples were centrifuged immediately at 2,500 g for 10 min at room temperature. Plasma was aliquoted into Eppendorf tubes, snap-frozen on dry ice, and stored at−80 °C until analysis.

Red blood cells were also aliquoted and stored at −80 °C until analysis.

### 2.5 Cytokine analysis

Myeloperoxidase (MPO), Interleukin-6 (IL-6), Interleukin-8 (IL-8), Interleukin-10 (IL-10), Tumor Necrosis Factor-alpha (TNF-α), C-C Motif Chemokine Ligand 2 (CCL-2)/Monocyte Chemoattractant Protein-1 (MCP-1) and Insulin-like Growth Factor Binding Protein (IGFBP) were quantified from 50-μL plasma samples in duplicates using a magnetic Luminex ELISA assay (Bio-Techne Ltd., Abingdon, Oxon, UK).

### 2.6 Hematological parameters

Hemoglobin concentration, hematocrit, and red blood cell count, mean corpuscular volume (MCV), mean corpuscular hemoglobin (MCH), mean corpuscular hemoglobin concentration (MCHC), and reticulocyte hemoglobin content were assessed with an automated hematology analyzer (Sysmex, Kobe, Japan).

### 2.7 Calculation of HS-Omega-3 Index and lipophilic index

Fatty acid analysis was conducted by Omegametrix GmbH (Munich, Germany). The high-sensitivity Omega-3 Index (HS-O3I) was calculated based on the fatty acid composition of erythrocyte membranes, specifically measuring 26 individual fatty acids using gas chromatography with flame ionization detection (GC-FID) under defined analytical conditions, as previously described (23).

The lipophilic index (LI) was then calculated by weighting the melting points of the detected fatty acids by their relative abundance in the erythrocyte membrane, following previously published approaches. Melting point data were obtained from a curated Japanese fatty acid database that has been used in several peer-reviewed studies and is considered a reliable and comparable reference source (24, 25).

For the LI calculation, only fatty acids included in prior publications were considered. Consequently, five fatty acids (elaidic acid, eicosadienoic acid, dihomo-γ-linolenic acid [DGLA], adrenic acid, and docosapentaenoic acid [DPA, n-6]) were excluded, as no reliable melting point data were available for four of them. The fatty acids included in the LI calculation covered on average 93.4% of the total erythrocyte fatty acid composition, which is consistent with the ~92% reported by Wu et al. (25). Thus, the exclusion of these fatty acids is unlikely to have influenced the LI calculation in a meaningful way.

### 2.8 Hormonal parameters

Hormonal parameters, including testosterone (measured once during the early follicular phase), follicle-stimulating hormone (FSH), luteinizing hormone (LH), estradiol, and progesterone, were measured using chemiluminescent immunoassay (CLIA) techniques by an accredited medical laboratory (SYNLAB Medical Care Center, Bad Nauheim, Germany).

### 2.9 Football specific performance tests

Linear sprint performance was assessed using a 30 m sprint test, which is a widely applied method in soccer performance diagnostics. Athletes performed the test on a natural grass pitch wearing their regular soccer footwear. Timing gates (single-beam light barriers) were positioned at 5 m, 10 m, and 30 m, with the first gate placed 1 m from the starting line, in accordance with standardized testing protocols (DFB guidelines). This setup enabled the assessment of both acceleration and maximal sprinting capacity.

Participants started in a standing position (high start) 1 m behind the first timing gate. To exclude reaction time as a potential confounding factor, no external start signal was given. Each athlete completed three maximal sprint trials over the 30 m distance, interspersed with 3 min of passive recovery to minimize fatigue effects. The best trial performance (fastest split times and overall 30 m time) was used for further analysis.

Explosive lower-limb power was assessed using the countermovement jump (CMJ), a widely applied test in soccer performance diagnostics due to its relevance for sport-specific explosive actions such as sprinting and jumping (26). Testing was conducted under standardized conditions following recommendations of established test batteries in soccer diagnostics (i.e., DFB test protocol). Each participant performed one familiarization trial followed by two recorded attempts. Athletes were instructed to keep their hands on their hips throughout the jump to eliminate the influence of arm swing, and jumps were deemed invalid if the hands left the hips or if an additional knee tuck was performed during flight. Between trials, athletes rested passively for 3 min to minimize fatigue and ensure maximal effort in each attempt. Jump height was recorded via contact mat measurement, which has been shown to provide reliable and valid assessments of vertical jump performance in applied sports settings (27). For further analyses, the best performance (highest jump height) out of the two valid trials was used, consistent with recommendations to improve measurement reliability.

Endurance capacity was assessed using a standardized progressive exercise test on a 400-m outdoor running track, as previously described by Kindermann et al. (28). In brief, participants started at a running speed of 8 km·h^−1^, which was increased by 2 km·h^−1^ every 3 min until volitional exhaustion. To ensure accurate pacing, subjects adjusted their running speed according to an audio signal. The test was terminated when participants were unable to reach a predefined mark at the corresponding pacing signal. At the end of each stage, subjects paused for 30 s to allow capillary blood sampling (20 μL) from the earlobe for lactate determination, and heart rate was simultaneously recorded using heart rate monitors (Polar, Büttelborn, Germany). Blood lactate concentrations were analyzed using a lactate analyzer (EKF Diagnostic Sales GmbH, Magdeburg, Germany). The individual anaerobic threshold (IAS) was calculated with Ergonizer software (Ergonizer, Freiburg, Germany), which defines the IAS as the lactate concentration at the individual lactate threshold plus 1.5 mmol·L^−1^ (29). The following parameters were obtained: maximum running speed, running speed at individual anaerobic threshold, running speed and heart rate corresponding to lactate concentrations of 2 and 4 mmol·L^−1^, peak lactate concentration, and maximal heart rate. The 2 and 4 mmol·L^−1^ fixed lactate thresholds were used to characterize exercise intensity at lower and higher submaximal levels, respectively.

### 2.10 Statistical analysis

To minimize bias, all biochemical and performance data were analyzed in a blinded manner. Laboratory staff and data analysts were unaware of group allocation during sample processing and statistical evaluation.

All statistical analyses were performed using JASP (Version 0.17.2.1, University of Amsterdam, Netherlands) and R version 4.5.1 (47) using Positron version 2025.09.0 (48). Data were inspected for normality and homogeneity of variance prior to analysis. Unless otherwise specified, all tests were two-tailed with an α-level set at 0.05.

Given the exploratory nature of this study and the range of secondary outcomes examined, no correction for multiple comparisons was applied. Results for secondary outcomes should therefore be interpreted as hypothesis-generating rather than confirmatory.

Descriptive statistics are presented by group and time point, with between-group differences assessed using Welch's *t*-test. For the primary outcome (omega-3 index), a two-tailed test was applied based on the *a priori* hypothesis that the intervention would increase HS-O3I. To examine the effects of the intervention on the omega-3 index, a 2 (Group: Intervention vs. Reference) × 3 (Time: Pre, Mid, Post) mixed-design ANOVA was conducted, with Group specified as the between-subjects factor and Time as the within-subjects factor. The model included the Group × Time interaction. Given the non-randomized design, findings for outcomes showing pre-existing between-group differences were interpreted cautiously, with consideration given to the Group × Time interaction when evaluating differential changes over time. Sphericity of the within-subject factor Time was assessed using Mauchly's test. When the sphericity assumption was violated, Greenhouse–Geisser-corrected degrees of freedom and *p*-values were reported; otherwise, uncorrected values are presented. *Post-hoc* pairwise comparisons with Holm adjustment were applied to further explore significant main effects of Time. To assess the predictive value of HS-O3I for physical performance, a simple linear regression was performed with post-intervention HS-O3I as the independent variable and performance in the individual anaerobic threshold (IAS, km/h) as the dependent variable. Associations between LI and HS-O3I were evaluated using Spearman correlation coefficients. For selected parameters, absolute change scores (delta - Δ) were calculated as Post minus Pre values to quantify intervention-related effects.

To investigate potential associations between HS-O3I levels and the presence of clinically relevant premenstrual symptoms (PMS), independent-samples *t*-tests were conducted, with PMS (yes/no) as the grouping variable and HS-O3I as the dependent variable. Menstrual and contraceptive status were controlled by study design through measurement timing standardization (mid-luteal or active pill phase) and were therefore not included as statistical covariates.

In cases where normality assumptions were not met, the non-parametric Mann-Whitney U test was employed. Effect sizes are reported as partial eta squared (ηp^2^) for ANOVA, Cohen's d for *t*-tests, and standardized beta coefficients (β) for regression analyses. Data are presented as mean ± standard deviation (SD) unless otherwise indicated.

## 3 Results

Baseline characteristics of participants by group are presented in Table 1. There were no significant differences between the intervention and reference groups in age, height, weight, or BMI at baseline (all *p* > 0.05).

**Table 1:** Baseline characteristics of participants by group.

Group	Age (years)	Weight (kg)	Height (cm)	BMI (kg/m^2^)
Reference	27.8 ± 4.8 (18)	65.2 ± 9.5 (18)	168.5 ± 5.9 (18)	22.9 ± 2.5 (18)
Intervention	25.3 ± 3.7 (20)	65.3 ± 8.2 (20)	167.9 ± 5.8 (20)	23.1 ± 2.2 (20)

Values are presented as mean ± SD.

Of the 38 enrolled athletes, 27 completed the full study protocol (14 in the DHA + whey group, 13 in the reference group), corresponding to an overall dropout rate of 29%. Reasons for dropout included scheduling conflicts, injury, or illness unrelated to the intervention. Dropout rates were comparable between groups (30% vs. 28%), indicating no differential attrition bias.

Key primary and secondary blood-based markers between intervention and reference group across all three time points are displayed in Table 2.

**Table 2:** Mean ± SD for selected blood-based outcomes with group-based comparisons at Pre, Mid, and Post assessments.

Outcome [95% CI] (*n*)	Intervention *Pre*	Intervention *Mid*	Intervention *Post*	Reference *Pre*	Reference *Mid*	Reference *Post*	P *Pre*	P *Mid*	P *Post*
HS-Omega-3 Index (%)	5.28 ± 1.20 [4.72, 5.84] (*n* = 20)	8.05 ± 1.73 [7.09, 9.01] (*n* = 15)	9.14 ± 1.85 [7.97, 10.31] (*n* = 12)	4.86 ± 1.33 [4.20, 5.52] (*n* = 18)	4.94 ± 1.41 [4.21, 5.66] (*n* = 17)	4.93 ± 1.55 [4.08, 5.79] (*n* = 15)	0.313	< 0.001	< 0.001
Lipophilic Index	18.91 ± 1.04 [18.42, 19.40] (*n* = 20)	18.49 ± 1.00 [17.94, 19.05] (*n* = 15)	18.65 ± 0.97 [18.04, 19.27] (*n* = 12)	18.97 ± 0.91 [18.52, 19.42] (*n* = 18)	18.83 ± 1.11 [18.26, 19.41] (*n* = 17)	19.35 ± 1.09 [18.74, 19.95] (*n* = 15)	0.855	0.368	0.094
Reticulocyte Hb (pg)	33.7 ± 0.9 [33.3, 34.1] (*n* = 19)	33.7 ± 1.1 [33.1, 34.3] (*n* = 15)	34.0 ± 1.0 [33.4, 34.6] (*n* = 12)	32.7 ± 1.4 [32.1, 33.4] (*n* = 18)	32.7 ± 0.9 [32.3, 33.2] (*n* = 17)	33.3 ± 0.7 [32.9, 33.6] (*n* = 15)	0.019	0.008	0.043
MCV (fL)	91.3 ± 3.8 [89.5, 93.1] (*n* = 20)	91.3 ± 3.3 [89.5, 93.1] (*n* = 15)	91.2 ± 2.8 [89.4, 92.9] (*n* = 12)	88.2 ± 2.2 [87.1, 89.3] (*n* = 18)	87.8 ± 2.6 [86.5, 89.2] (*n* = 17)	88.6 ± 1.7 [87.6, 89.5] (*n* = 15)	0.004	0.003	0.011
MPO (ng/mL)	6,603 ± 2,674 [5,314, 7,892] (*n* = 19)	6,071 ± 2,222 [4,788, 7,354] (*n* = 14)	5,371 ± 2,418 [3,835, 6,908] (*n* = 12)	10,162 ± 3,927 [8,210, 12,115] (*n* = 18)	9,522 ± 3,280 [7,835, 11,208] (*n* = 17)	10,254 ± 3,955 [8,064, 12,445] (*n* = 15)	0.003	0.002	< 0.001

### 3.1 HS-Omega-3 Index and lipophilic index

All blood and performance assessments were successfully aligned with the mid-luteal or active pill phase as planned. There was a significant main effect of time for the HS-O3I, (F(2, 48) = 87.593, *p* < 0.001, ηp^2^ = 0.79), indicating that scores changed significantly across the three measurement points (Pre: 5.08% ± 1.26%; Mid: 6.36 ± 2.21%; Post: 6.8 ± 2.7%). Moreover, there was a significant time × group interaction (F(2, 48) = 80.700, *p* < 0.001, ηp^2^ = 0.72) suggesting that the pattern of change over time differed between the intervention and reference groups. There was also a significant main effect of group (F(1, 24) = 17.689, *p* < 0.001, ηp^2^ = 0.42) indicating overall differences between the intervention and control groups. *Post-hoc* analyses revealed that the mean score significantly increased from Pre to Mid (t = −10.167, *p* < .001) and from Pre to Post (t = −12.423, *p* < 0.001). In addition, there was a statistically significant increase from Mid to Post (t = −2.256, *p* < 0.05) (Figure 3A).

**Figure 3:**
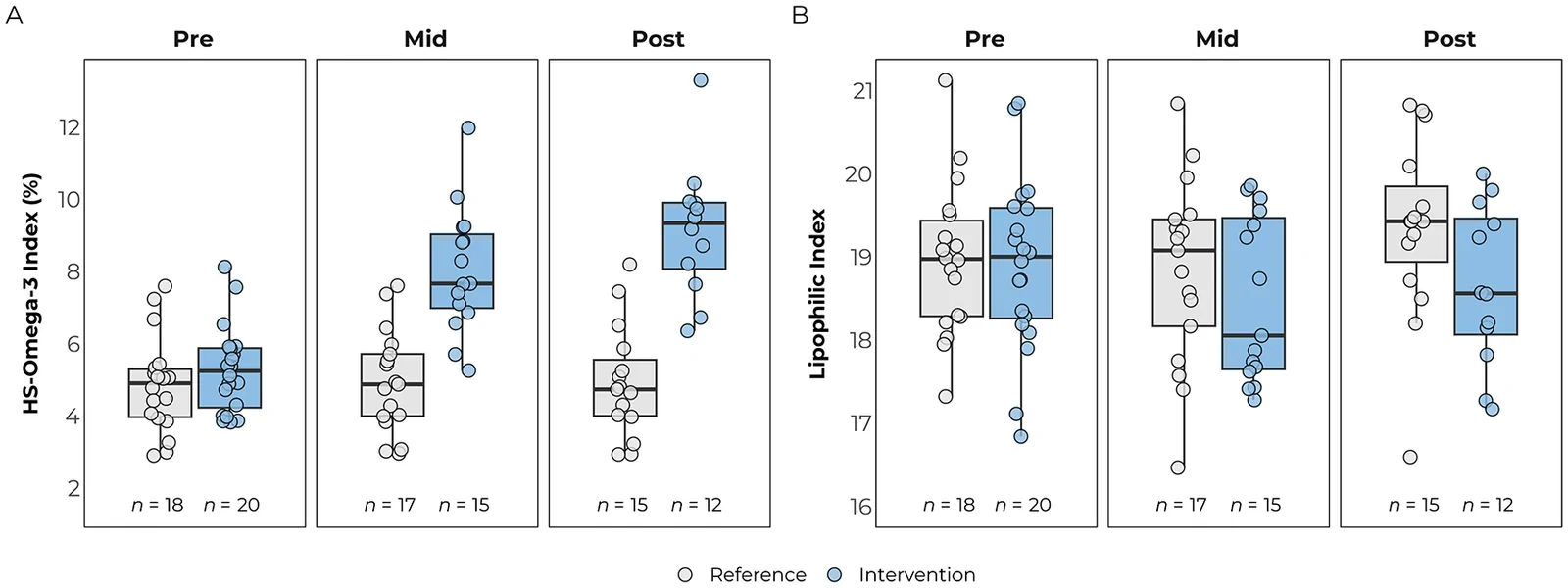
Changes of the HS-Omega-3 Index **(A)** and lipophilic index **(B)** over time within the reference and intervention group.

Importantly, dietary intake of omega-3 fatty acids, as assessed by the n-3 FA FFQ did not change between pre and post, except for the administered supplementation in the intervention group. This indicates that the observed increase in the HS-O3I was attributable to the supplementation rather than to alterations in habitual dietary intake.

The LI showed a significant main effect of time (F(2, 48) = 4.209, *p* < 0.05, ηp^2^ = 0.15) indicating that the LI changed significantly across the three measurement points. However, the time × group interaction was not statistically significant (F(2, 48) = 1.875, *p* > 0.05, ηp^2^ = 0.07) suggesting that the pattern of change over time did not differ significantly between the intervention and control groups. There was no significant main effect of group (F(1, 24) = 0.357, *p* > 0.05, ηp^2^ = 0.02) indicating that the intervention and control groups did not differ significantly in their overall levels of lipophilic index. *Post-hoc* comparisons revealed a significant increase in LI from Pre to Mid (t = 2.411, *p* < 0.05) and a significant decrease from Mid to Post (t = −2.603, *p* < 0.05). No significant difference was found between Pre and Post (t = −0.192, *p* > 0.05) (Figure 3B).

A significant moderate negative correlation was observed between the HS-O3I and LI at Pre (r = −0.45, *p* = 0.005) (Figure 4A). A moderate negative correlation was also observed between the Delta of LI and HS-O3I (r = −0.57, *p* < 0.005) (Figure 4B).

**Figure 4:**
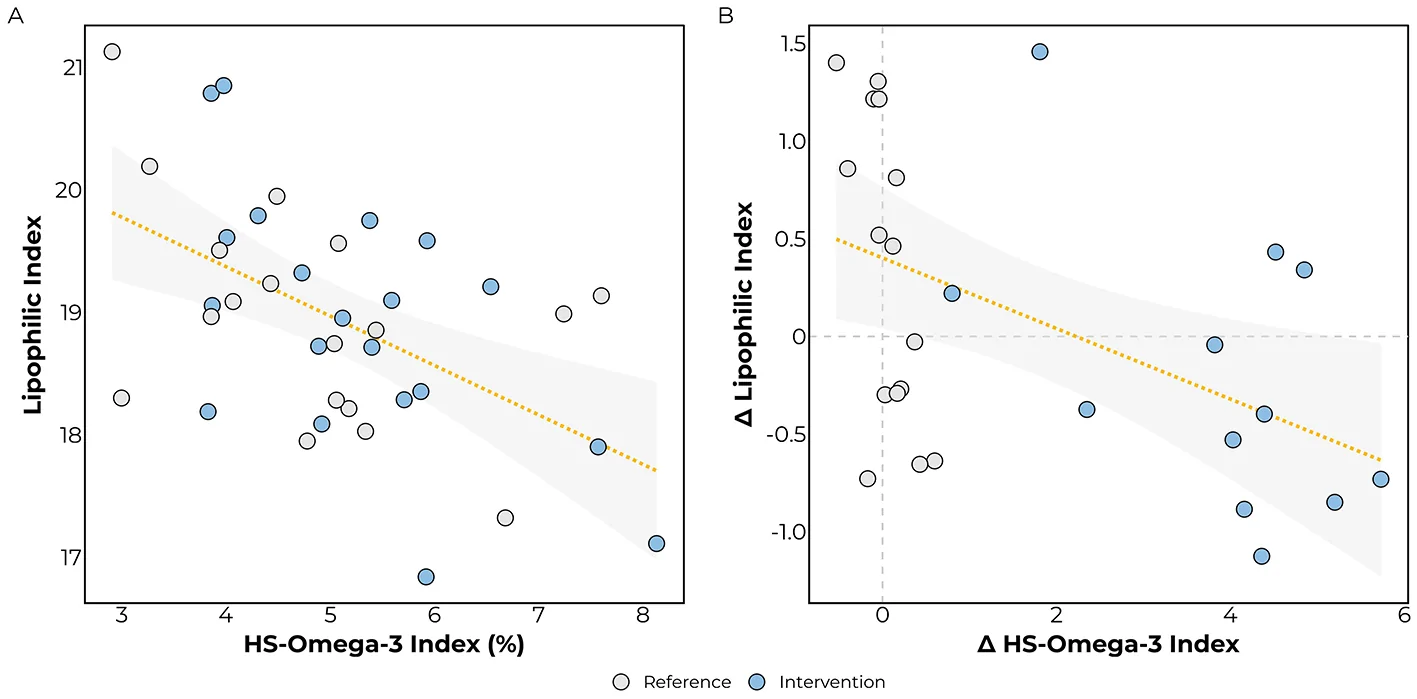
Correlation between HS-Omega-3 Index and lipophilic index at Pre **(A)** and HS-Omega-3 Index delta and Lipophilic Index delta **(B)**.

### 3.2 Hematological parameters

Analysis of the hematological parameters revealed no significant changes over time for erythrocytes, hematocrit, hemoglobin, MCH, MCHC, or reticulocyte percentage (all *p* > 0.05).

For mean corpuscular volume (MCV), no significant main effect of time was found; however, a significant group × time interaction was observed (*p* < 0.05, ηp^2^ = 0.13), indicating that the temporal pattern of MCV differed between groups. In addition, there was a significant main effect of group (*p* < 0.005, ηp^2^ = 0.37), indicating that MCV differed significantly between the intervention and control groups across the study period (Figure 5A).

**Figure 5:**
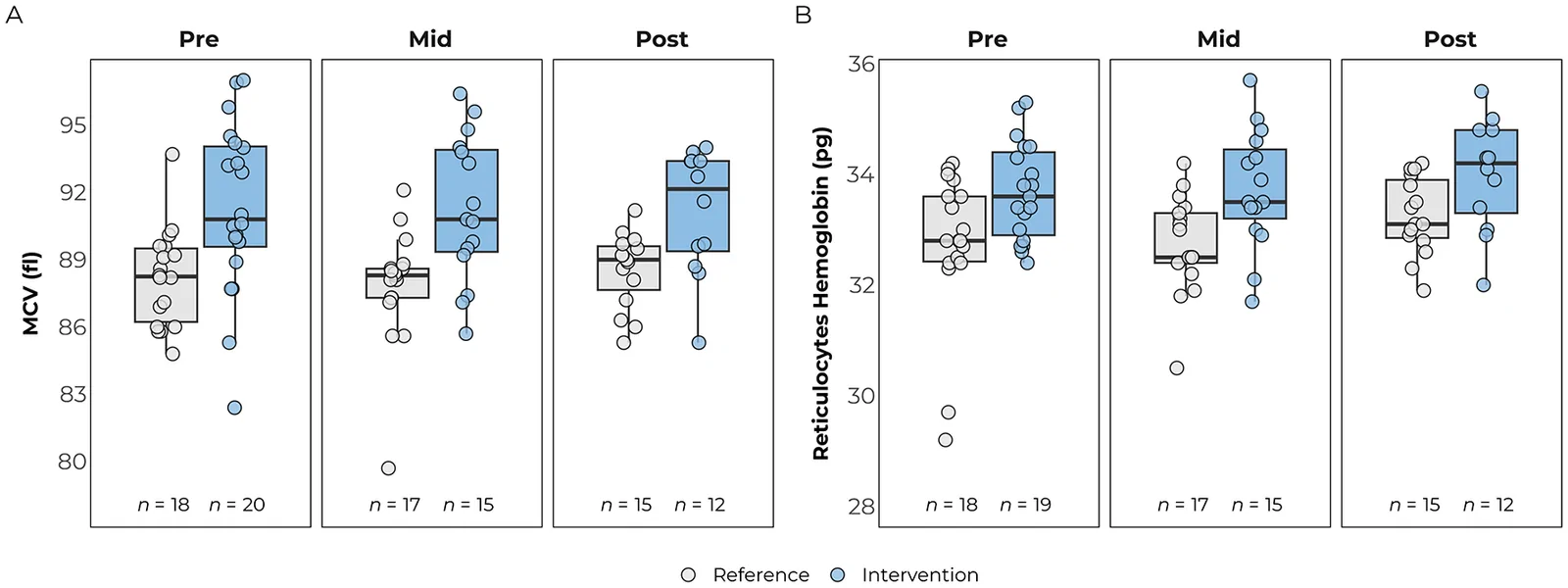
Changes of mean corpuscular volume **(A)** and reticulocyte hemoglobin **(B)** over time within the reference and intervention group.

Regarding reticulocyte hemoglobin content, no significant time effect or group × time interaction was detected. Nevertheless, a significant main effect of group was present (*p* < 0.005, ηp^2^ = 0.33), indicating a persistent difference between the intervention and reference groups across the study period. Notably, this difference was already apparent at Pre (mean ± SD: control 32.74, intervention 33.68; *p* < 0.05) (Figure 5B).

### 3.3 Cytokines

There was a significant main effect of time, (F(2, 46) = 5.144, *p* < 0.05) indicating that MPO values changed over time across all participants. However, the time × group interaction was not significant, (F(1, 24) = 1.195, *p* > 0.05), suggesting that the pattern of change over time did not differ significantly between the intervention and reference group.

A significant main effect of group was found, (F(1, 23) = 9.248, *p* < 0.05), indicating that overall MPO levels differed significantly between the intervention and reference groups, irrespective of timepoint (Figure 6). No significant effects were observed for IL-6, IL-8, IL-10, TNF-α, CCL-2/MCP-1, or IGFBP (all *p* > 0.05).

**Figure 6:**
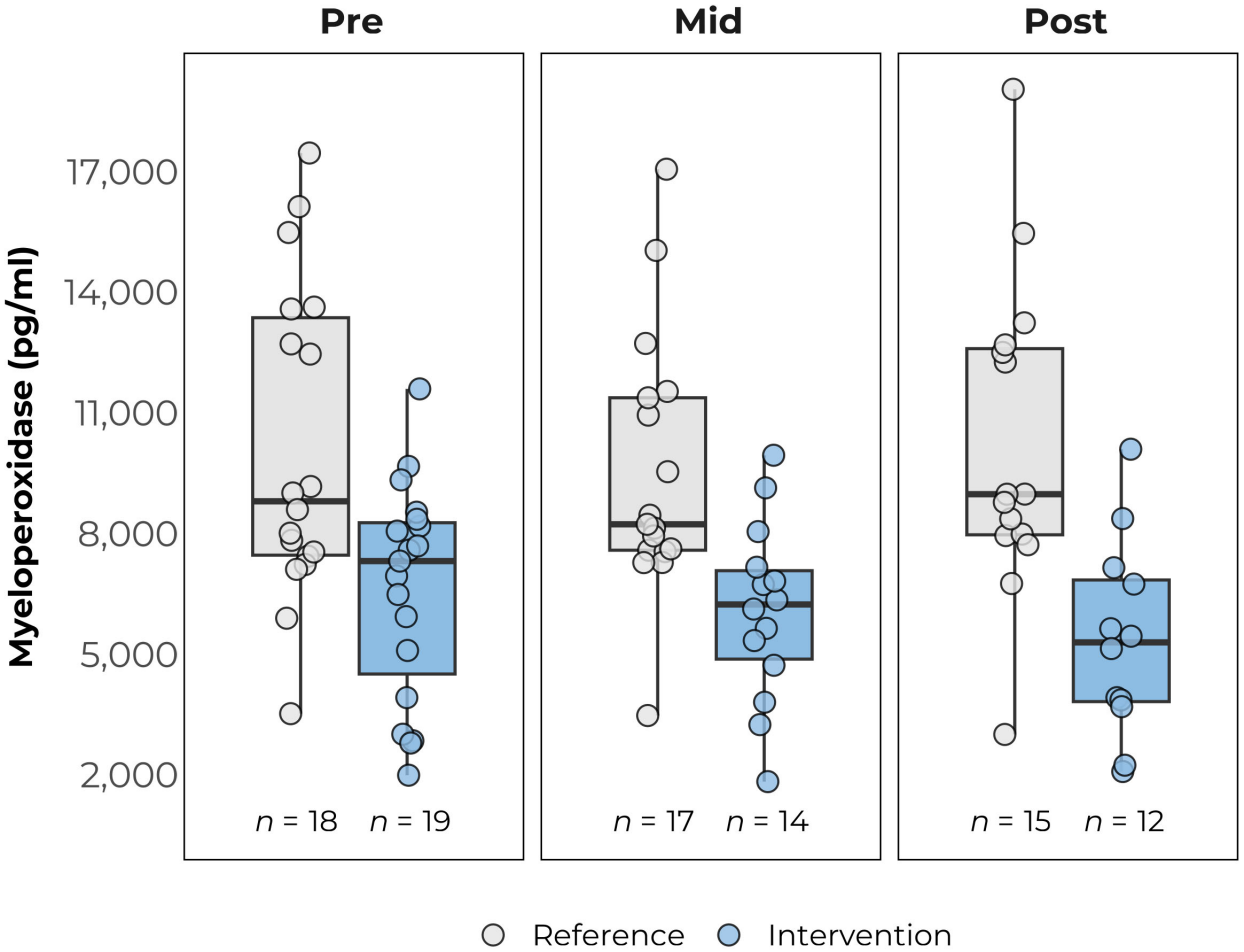
Changes of myeloperoxidase over time within the reference and intervention group.

### 3.4 Menstrual symptoms and hormonal profiles

13 of the 27 athletes that completed the full study protocol were naturally cycling and 14 used oral contraceptives.

The mean HS-O3I in the non-PMS group (4.96% ± 1.19) did not differ significantly from the PMS-group (5.45%, SD ± 1.48) (U = 109.00, *p* > 0.05).

Further no changes in female specific hormones (estrogen, progesterone, LH and FSH) displayed across time points (*p* > 0.05).

### 3.5 Associations between HS-Omega-3 Index and performance variables

19 individuals conducted the performance tests. No time effect for sprint, jump or individual anaerobic threshold was found. And further there was no significant time x group effect. However, an association was found between HS-O3I and endurance performance (individual anaerobic threshold - IAS). The overall regression model for Post was significant (F(1, 17) = 9.434, *p* < 0.05) indicating that the HS-O3I accounted for a significant proportion of the variance in IAS performance. The model explained 35.7% of the variance in performance (adjusted R^2^ =.319). The regression model indicates that for each one-unit increase in the HS-O3I, IAS performance increased by approximately 0.33 km/h (b = 0.327, SE = 0.106, 95% CI [0.103, 0.551], t = 3.072, *p* < 0.05).

## 4 Discussion

This nutritional intervention study investigated the effects of 4 months of encapsulated algae-based DHA in a protein formulation in semi-professional female soccer players on the HS-O3I, hematological parameters, inflammatory status, performance, and menstrual health. Our main finding, in line with previous reports (1), was that baseline omega-3 status in female athletes was suboptimal according to the proposed reference range. Daily supplementation with 1,000 mg DHA significantly improved omega-3 levels, with marked increases already detectable after 2 months. In parallel, changes in the LI indicated structural remodeling of erythrocyte membranes. On the hematological level, the intervention group displayed higher mean MCV, and reticulocyte hemoglobin content compared to the reference group. No group effects were observed for cytokines, except for MPO, which decreased over time. Furthermore, no significant effects were found on premenstrual symptoms or performance.

### 4.1 Omega-3 status and erythrocyte remodeling

The suboptimal baseline omega-3 status in this cohort confirms previous findings in athletes, where mean values often fall below the recommended target of 8–11% (12). This underscores the importance of targeted nutritional strategies to improve long-chain omega-3 PUFA intake in athletic populations. The robust improvement of the HS-O3I following DHA supplementation highlights the responsiveness of erythrocyte fatty acid composition to dietary intake. These improvements have been shown in several studies (30–33) but the link to performance and the benefit of this increase of PUFA levels has been only inconsistently described. Therefore, this study investigated how this improves parameters of athletic health and performance.

The observed increase in the LI within the first 2 months of supplementation, followed by stabilization, thereafter, may suggest a ceiling effect. This would be in line with the notion that the fatty acid composition of erythrocyte membranes is tightly regulated to preserve optimal membrane properties (34). Once a critical threshold of incorporation is reached, further increases in omega-3 content may be restricted to avoid destabilizing the balance of membrane lipids. Such regulation has functional relevance: erythrocyte deformability depends on an optimal range of membrane fluidity. Both insufficient and excessive unsaturation could compromise structural integrity (35). Here we must ask the question what the adequate supplementation dose for athletes and physiological beneficial HS-O3I would be. The proposed reference range of von Schacky (12) applies to the clinical setting, but can this simply be transferred to the athletic population?

It should be noted, however, that erythrocyte deformability was not directly measured in the present study; the following mechanistic considerations are therefore based on indirect evidence from membrane composition changes. Erythrocyte deformability is the ability of RBC to reversibly change their shape during flow in response to external forces. This property allows them to pass through narrow microvessels while transporting O_2_ and CO_2_ to tissues and cells in the body (36). Improved RBC deformability is particularly important in endurance sports, where erythrocytes are exposed to repeated mechanical and oxidative stress. Under these conditions, erythrocytes are prone to hemolysis, like foot strike hemolysis in runners (37). More resilient membranes, supported by balanced remodeling, may help mitigate these losses and sustain efficient oxygen delivery during prolonged exercise.

### 4.2 Hematological findings

In line with the membrane remodeling indicated by the LI, additional alterations were observed on the hematological level. In our study, athletes receiving DHA+whey displayed higher MCV and reticulocyte hemoglobin compared to controls.

While the interpretation of these parameters requires caution, some mechanistic considerations can be made. As reticulocytes are the direct products of erythropoiesis, they provide an almost immediate snapshot of iron availability. Because they mature within a few days, their hemoglobin content is not diluted by older erythrocytes that were produced under different iron conditions. While the reticulocyte count itself is a robust parameter for the quantity of erythropoiesis, hemoglobinization of reticulocytes adds important information on the quality of red cell production, directly reflecting the adequacy of iron supply during hemoglobin synthesis (38).

Increased MCV, on the other hand, is typically associated with larger erythrocyte size. In the context of endurance training, this may reflect a shift toward a younger red cell population (reticulocytosis) (39). These mechanisms could potentially influence blood rheology and oxygen transport dynamics, although direct evidence for a performance benefit is still lacking.

Taken together, the observed differences in MCV and reticulocyte hemoglobin may be compatible with subtle differences in erythrocyte production or structure. However, they do not establish supplementation-induced hematological adaptations, particularly because the reticulocyte hemoglobin difference was already apparent at baseline. Moreover, because the intervention combined DHA with whey protein and the reference group received no comparator product, the observed hematological differences cannot be attributed specifically to DHA and may reflect the combined intervention with the protein component. The differential temporal pattern observed for MCV remains an exploratory finding that warrants confirmation in randomized studies using matched comparator conditions and incorporating direct measures of erythrocyte turnover, membrane lipidomics, and hemorheological properties.

### 4.3 Inflammatory and menstrual health outcomes

In the present study, no consistent effects of DHA supplementation were observed on systemic cytokine response or on the severity of premenstrual symptoms. This contrasts with findings in clinical and patient populations, where omega-3 supplementation has repeatedly been associated with reductions in systemic inflammation (40–42) and improvements in menstrual health outcomes (10). Although all assessments were scheduled according to reproductive status, during the mid-luteal phase in naturally cycling athletes and during the active-pill phase in oral-contraceptive users, both groups were pooled in the statistical analyses. The distinct hormonal profiles of naturally cycling athletes and oral-contraceptive users may therefore have obscured subgroup-specific or hormone-sensitive responses. Future studies should examine these groups separately and use repeated symptom assessments to determine whether omega-3 supplementation influences PMS severity over time.

The lack of broad effects in our cohort likely reflects the overall healthy and well-trained status of semi-professional athletes, who typically display low baseline inflammation and more cycle-related symptoms during the first days of their menstrual cycle (43), thereby limiting the potential for detectable improvements. Nevertheless, in parallel to the membrane remodeling and hematological alterations observed in the intervention group, we detected a subtle decline in MPO levels over time. MPO is a marker of neutrophil activation and oxidative stress, both of which are accentuated during repeated high-intensity training loads (44). A reduction in MPO may therefore represent an early sign of attenuated immune activation or improved redox balance, potentially supporting recovery and resilience in demanding training environments. While this effect did not translate into overt performance benefits within the study period, it may fall in line with the observed hematological alterations by suggesting that omega-3 supplementation may confer broader systemic stability at the interface of erythrocyte function, oxidative stress, and immune regulation (45).

In our study, the supplementation protocol provided 1,000 mg of DHA per day without additional EPA. Experimental evidence indicates that EPA and DHA may exert distinct, though partially overlapping, effects on inflammatory signaling. For instance, controlled cell culture and human studies have shown that EPA and DHA differentially modulated LPS-stimulated monocyte cytokine expression: EPA primarily lowered TNF-α, whereas DHA reduced TNF-α, IL-6, MCP-1, and IL-10, indicating a broader immunomodulatory profile of DHA. Both fatty acids altered the plasma specialized pro-resolving mediator lipidome, but DHA induced a wider shift toward anti-inflammatory derivatives, suggesting potentially stronger resolution-phase effects (42).

However, findings from a network meta-analysis of randomized controlled trials indicate that DHA and EPA have largely comparable effects on systemic markers of subclinical inflammation, including C-reactive protein (CRP), IL-6, and TNF-α. Collectively, these findings imply that while mechanistic differences between EPA and DHA exist at the cellular level, such distinctions may not consistently translate into detectable systemic anti-inflammatory effects, particularly at moderate doses such as those applied in the present study (46).

### 4.4 HS-Omega-3 Index improvements and performance

Despite the robust increases in the HS-O3I observed after DHA supplementation, no direct effects on sprint performance, countermovement jump height, or endurance capacity were detected in this study. This missing link between biochemical changes and physical performance suggests that improvements in omega-3 status do not immediately translate into functional benefits. Nevertheless, the concurrent remodeling of erythrocyte membranes, as indicated by changes in the lipophilic index, provides indirect evidence that omega-3 incorporation alters structural properties relevant to oxygen transport. Improved erythrocyte deformability, which has been linked to enhanced microvascular perfusion and tissue oxygenation (36), may constitute a physiological mechanism through which elevated omega-3 status could support endurance performance. That could also be the reason why we found an association between the HS-O3I and endurance performance. While such adaptations may not immediately manifest in standardized field tests, they could contribute to resilience under sustained or extreme exercise conditions, where efficient oxygen delivery and reduced hemolysis are critical.

In this context, our findings should be interpreted as preliminary and hypothesis-generating, providing a mechanistic rationale for performance effects that remains to be confirmed in future work. Future studies should integrate more direct physiological assessments, such as measures of blood rheology, *in vivo* oxygen extraction, or muscle metabolism during exercise. Such approaches would allow testing of the hypothesis that higher HS-O3I values confer functional advantages by optimizing erythrocyte properties and oxygen transport dynamics.

### 4.5 Limitations

Several limitations of this study should be acknowledged. Endurance performance was assessed using field-based tests rather than laboratory-based cardiopulmonary exercise testing. While this approach increased ecological validity and better reflected sport-specific demands, it limited the precision in quantifying oxygen transport and utilization, potentially masking subtle physiological adaptations.

Due to the combined administration of DHA and whey protein, it is not possible to definitively attribute the observed effects to DHA alone, protein intake, or the synergistic interaction of both. Thus, the independent contribution of each component cannot be clearly distinguished.

Another limitation concerns the supplementation protocol, which included DHA alone without additional EPA. Given the partly distinct biological actions of EPA and DHA, the absence of EPA may have attenuated potential anti-inflammatory and performance-related effects and limits the generalizability of our findings to combined n-3 PUFA interventions.

Furthermore, the absence of random allocation, implemented to enhance adherence and accommodate dietary preferences may have introduced selection bias that cannot be fully ruled out. For the same reason, no placebo was administered to the reference group, as this was considered ethically unjustified and logistically not feasible given the preference-based allocation. In addition, the relatively high dropout rate and modest final sample size may have reduced statistical power to detect smaller but potentially meaningful effects.

This study also did not include a formal intention-to-treat analysis with imputation of missing values; all analyses were conducted on an available-case basis. Combined with the relatively high dropout rate (29%) and the non-randomized allocation, this raises the possibility of attrition-related bias that cannot be fully excluded. While dropout rates were comparable between groups (30% vs. 28%) and baseline data in Table 1 reflect nearly the full enrolled cohort, a formal statistical comparison of completers vs. non-completers on baseline characteristics was not performed.

Finally, no global correction for multiple testing was applied across the broad range of secondary outcomes. Consequently, the risk of type I error is increased, and isolated findings concerning hematological, inflammatory, hormonal, and performance-related outcomes should be interpreted as exploratory and hypothesis-generating.

## 5 Conclusion

This study contributes further evidence that 4 months of DHA supplementation in semi-professional female soccer players robustly increases the HS-O3I and may have induced remodeling of erythrocyte membranes. These findings extend previous work conducted predominantly in clinical populations by demonstrating that even in young, healthy, and well-trained female athletes, omega-3 status is modifiable and accompanied by exploratory differences in selected hematological parameters, although these differences cannot be attributed specifically to DHA. Importantly, however, these biochemical changes did not translate into measurable improvements in performance or menstrual health outcomes within the study period. Here, it might be that secondary effects may occur with a delay. Beyond these primary findings, MPO levels, a marker of neutrophil activation and oxidative stress, differed significantly between groups and changed significantly over time, suggesting a possible link between omega-3 supplementation and attenuated immune activation. While preliminary and not directly assessed in relation to performance, this finding suggests that omega-3 incorporation may influence inflammatory tone in a more nuanced manner than can be captured by performance outcomes alone.

Our results therefore add to the evidence base by showing that the incorporation of DHA into erythrocyte membranes in an athletic population may represent a preliminary physiological step warranting further investigation. Rather than serving as an acute ergogenic aid, the combined DHA-plus-whey supplementation strategy may influence erythrocyte lipid composition and potentially interact with hematological and inflammatory processes, although these secondary relationships require confirmation in randomized, placebo-controlled studies.

Future research should explore whether combined EPA and DHA supplementation offers greater functional benefits than DHA alone and identify the omega-3 threshold at which erythrocyte adaptations enhance oxygen transport and performance. Investigating whether athletes with elevated inflammation or pronounced premenstrual symptoms experience amplified effects will also be valuable. To advance practical applications, studies should include cardiopulmonary diagnostics, isolate EPA and DHA effects, and test varied dosing strategies across diverse athlete populations and sport-specific contexts. These efforts will help clarify the potential of omega-3 optimization as a targeted strategy to support athletic performance and recovery.

## Data Availability

The raw data supporting the conclusions of this article will be made available by the authors, without undue reservation.
